# Telomere length as a function of age at population level parallels human survival curves

**DOI:** 10.18632/aging.202498

**Published:** 2021-01-11

**Authors:** Paolina Crocco, Francesco De Rango, Serena Dato, Giuseppina Rose, Giuseppe Passarino

**Affiliations:** 1 Department of Biology, Ecology and Earth Science, University of Calabria, Rende 87036, Italy

**Keywords:** telomere, mortality deceleration, aging, lifespan

## Abstract

Telomeres are subject to age related shortening which can be accelerated by oxidative stress and inflammation. Many studies have reported an inverse correlation between telomere length and survival, but such inverse correlation has not been always confirmed in different populations. We analyzed the trend of Leukocyte Telomere Length (LTL) as a function of age in a cohort of 516 subjects aged 65-106 years from Southern Italy. The trend of LTL obtained was quite similar to demographic survival curves reported with data of western societies. We observed a decrease of LTL after 70 years of age and then an increase after 92 years, in agreement with the sharp decrease of survival after 70 years of age and its increase after 90 years, due to the deceleration of mortality at old ages. Our data suggest that a generalized LTL attrition after 70 years of age, associated to organismal decline, affects most of the population. Such generalized attrition may exacerbate senescence in these subjects, predisposing them to high mortality risk. Conversely, the subjects with better physical conditions, experience a lower attrition and, consequently, a delayed senescence, contributing to the deceleration of mortality which has been observed among very old subjects in modern societies.

## INTRODUCTION

Telomeres are specific structures that protect the ends of linear chromosomes from fusion and degradation.

Telomere length, which is usually studied in leucocytes (leucocytes telomere length, LTL), has been extensively studied in relation to aging and longevity as it has been reported to be inversely associated with age and with age-related decline. Indeed, short telomeres have been associated to numerous age associated diseases (such as cardiovascular diseases, neurodegenerative diseases, type 2 diabetes mellitus, premature aging syndromes, and cancers [1–5] (and references therein). Consistently, a study by Njajou et al [6] suggested LTL to be an informative biomarker of healthy aging. A similar result was reported by Kim et al [7] who observed a positive association of LTL with several measures of healthy aging in an age-dependent way. LTL was also found to correlate positively with physical ability in Danish twins aged 70+ [8]. Centenarians, considered a model of successful aging, have demonstrated to be generally characterized by longer telomeres, as well as their offspring show longer LTL with respect to people of the same age from the general population [9]. Further, the paper by Terry et al [10] have reported that healthy centenarians had significantly longer telomeres than did unhealthy centenarian.

Although it has often been correlated with life expectancy, conflicting data about LTL as a predictor of mortality in older adults have been reported. While some studies have found that LTL is a marker of mortality [1, 11–14], others have not found such association [6, 15–18], getting the relationship of LTL to aging and survival in humans unclear.

LTL is largely defined by genetic and environmental factors early in life [19, 20], and is substantially maintained during adulthood, although some lifestyles may significantly accelerate telomere shortening. Among the factors affecting LTL, oxidative stress and inflammation have been indicated. Consistently, smoking habits, drug abuse, obesity, sedentary life have been associated to shorter LTL [21–25]. As to the genetic factors, different estimates of the heritability for telomere length vary from 35 to 80% [9, 26, 27]. Genetic determinants of telomere length have been found in polymorphic variants at genes involved in leucocytes LTL maintenance, (like *TERT* and *TERC*), as well as in DNA repair mechanisms, (like *PARP1*, *ATM*, *POT1*), identified in candidate gene and genome wide association studies (GWAS) [28–35]. Telomere maintenance is strictly related to telomerase activity, a specialized ribonucleoprotein complex responsible for adding telomeric repeats to the ends of chromosomes. In most human somatic cells, except for stem cells and lymphocytes, telomerase activity gradually diminishes after birth. The level of telomerase activity is low in most adult somatic stem cells, whereas it is upregulated in some progenitor cell types and cancer cells [36]. Basically, the lack of telomerase activity is considered the main cause of physiological telomere shortening at each cell division and the leading contributor to replicative senescence [37–39].

In the aging subjects, attrition, and the consequent shortening, of telomeres undergo an acceleration. Berglund et al. [40] reported that the acceleration of telomere shortening starts at 69 years of age, with a significant inter-individual variation. Age related attrition of telomeres, and its acceleration, may be related to increase of oxidation and inflammation which are typical of old age and to the genetic background [9, 26, 27]. Different studies demonstrated that for a given age, about 60% of the inter-individual variation in LTL and 30% of its age-dependent attrition are heritable [26, 41]. Papers on this topic highlight the importance of long-term longitudinal studies and multiple measurements of LTL over time to have a more comprehensive picture of telomere length changes across the life course and its influence on lifespan [42–46]. But such studies are difficult to undertake in humans.

Given the different factors affecting telomere length (environmental and genetic factors in the first two decades, lifestyle in adulthood, genetic background, lifestyle and health status at old age) it is not surprising that the meaning of LTL in old subjects is difficult to be fully understood. Similarly, the contrasting results regarding the correlation between LTL and lifespan may reflect the overestimation of some of the different factors.

Here, to gain more insights into the relationship between LTL, survival and lifespan, we examined the association of LTL with all-cause mortality, using ten-years follow-up survival data of 516 subjects 65+ years of age, including centenarians. Furthermore, taking advantage of a second measurement after seven years in a sub-sample of individuals, we investigated the influence of telomere attrition on survival at very old age.

## RESULTS

**Figure 1:**
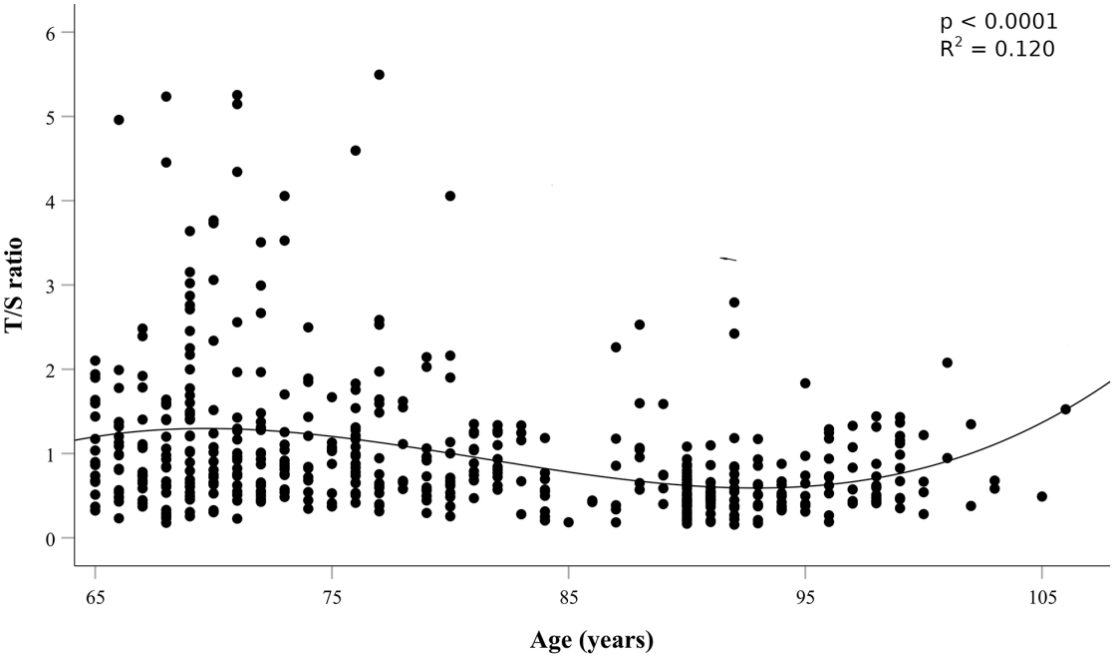
**Leukocyte Telomere Length (LTL) expressed as T/S ratio as a function of age.**

LTL expressed as the T/S ratio was measured in a sample of 516 subjects (240 males and 276 females; aged 65-106 years; median ages 80.54+10.87). The demographic characteristics of the whole sample and of the sample divided by age groups are provided in Table 1. The mean LTL value at baseline was 0.99 ± 0.82 T/S (range, 0.16–5.55, median 0.73), with no difference between males and females (Supplementary Figure 1; p>0.05). Figure 1 reports LTL distribution as a function of age.

**Table 1:** Demographic characteristics of the sample.

**Total sample (%)**			
***Age range (mean ± SD)***	***Male***	***Female***	***Total***
65-106 (80.54 ± 10.87)	240 (46.50)	276 (53.50)	516 (100)
**Distribution by age groups (%)**			
***Age range (mean ± SD)***	***Male***	***Female***	***Total***
65-82 (72.47 ± 4.98)	162 (31.40)	144 (27.90)	306 (59.30)
83-92 (89.33 ± 2.48)	44 (8.53)	82 (15.89)	126 (24.40)
93-106 (96.76 ± 2.98)	34 (6.59)	50 (9.69)	84 (16.30)
**Longitudinal subsample (%)**			
***Mean age (SD)***	***Male***	***Female***	***Total***
78.69 (3.85) at baseline			
85.33 (3.91) after 7 years	9 (25)	27 (75)	36 (100)

A progressive decrement in LTL is evident up to an inflection point around the age range 83 to 92 years, after which LTL increase slowly. This distribution was statistically significant (p<0.0001), that is the line that best captures the trend of the relationship between T/S ratios and age. Consistently, results of linear regressions showed: a significant negative correlation between T/S ratios and age in the whole sample (p<0.0001; Supplementary Figure 2A), a weak but negative correlation between 65 to 82 years (p=0.08; Supplementary Figure 2B); no correlation between 83 to 92 years (p=0.277; Supplementary Figure 2C), a significant positive correlation between 93 to 106 years (p= 0.003; Supplementary Figure 2D).

No correlation between survival probability and LTL was found (p=0.673).

**Figure 2:**
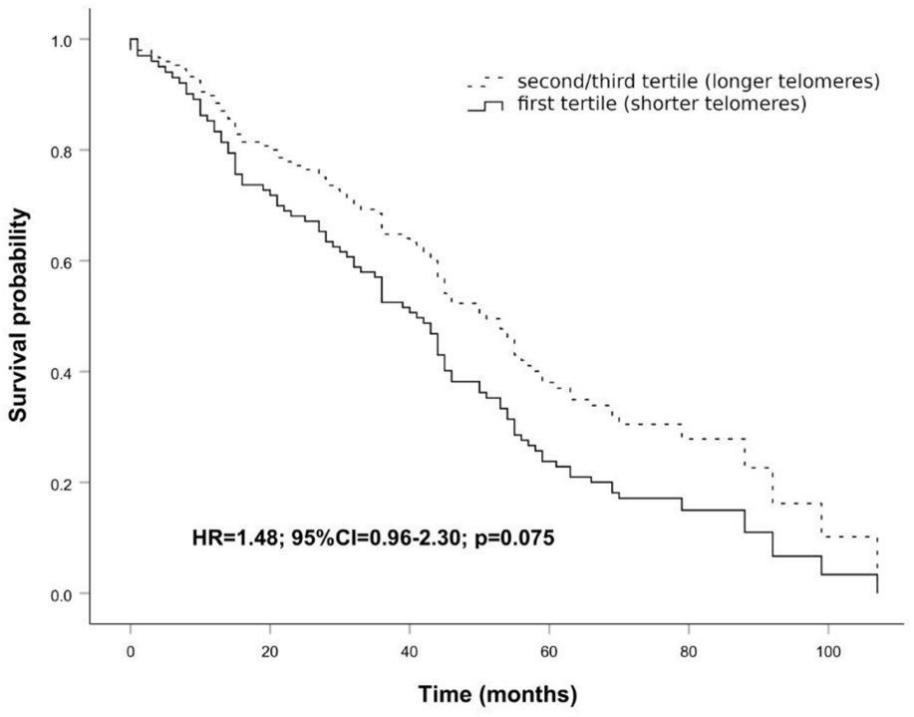
**Survival functions relative to carriers of shorter telomeres (first tertile) vs carriers of longer telomeres (second/third tertile) conducted on the age range 83 to 92 years.** The Cox regression was adjusted for age and sex. Time is expressed in months, where zero is considered the time of recruitment. HR value, confidence interval, and p-value from Cox regression analysis are reported inside the figure.

The peculiar LTL distribution curve of the cohort shown in Figure 1 prompted us to investigate the survival probability in the age window from 83 to 92 years. As it can be seen in survival curves in Figure 2, individuals within this age range having longer telomeres (second and third tertiles) seem to live longer, about eleven months, than those having shorter ones (first tertile), although this difference is not significant (HR = 1.48; 95% CI = 0.96-2.30; p = 0.075). This result may suggest that telomere length could affect mortality risk in this age window.

### Longitudinal study

For 36 subjects (Table 1) we had available two LTL evaluations, at baseline (mean age 78.69+3.85) and after 7-years of follow-up when their mean age was 85.33+3.91, falling in the above age-window. This allowed us to address the impact of telomere attrition in determining the risk of mortality.

**Figure 3:**
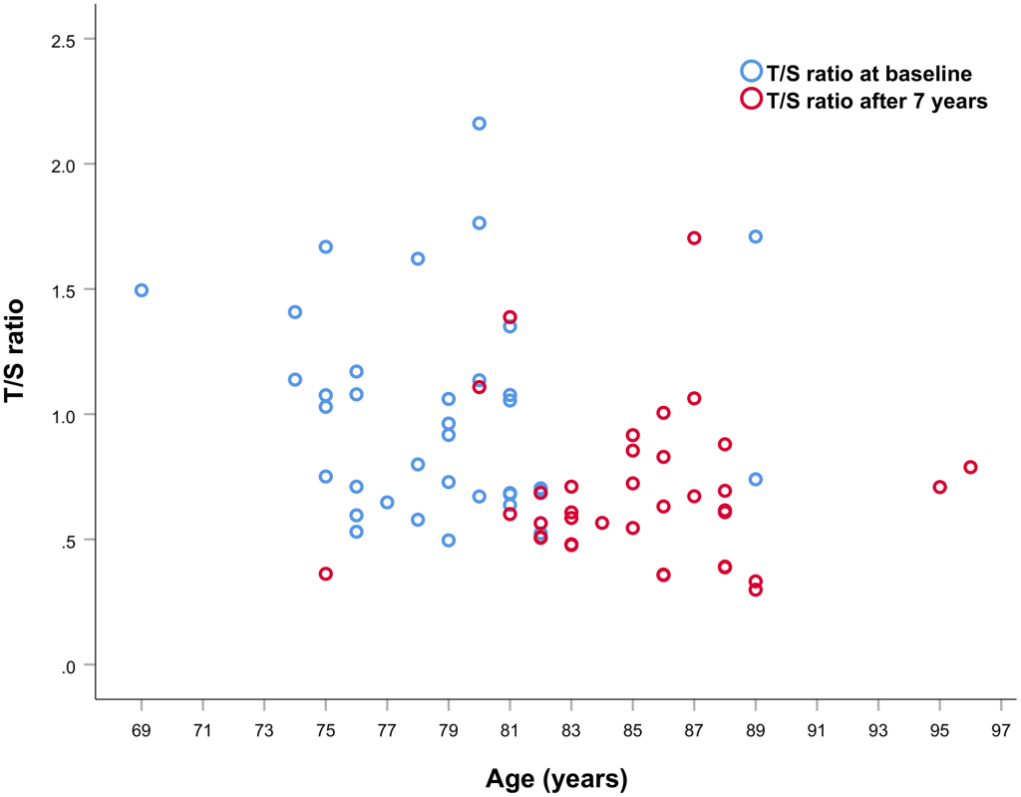
**Scatter plot of T/S ratio values at first and second sampling of the 36 subjects analyzed.**

A decrease of about 13.64% in LTL between the first and second sampling was observed (mean ± SD LTL at baseline 1.00+0.41; mean ± SD LTL at follow-up 0.88+59. p = 0.048), as it emerges from scatter plot of the T/S ratios at baseline and after 7 years showed in Figure 3.

**Figure 4:**
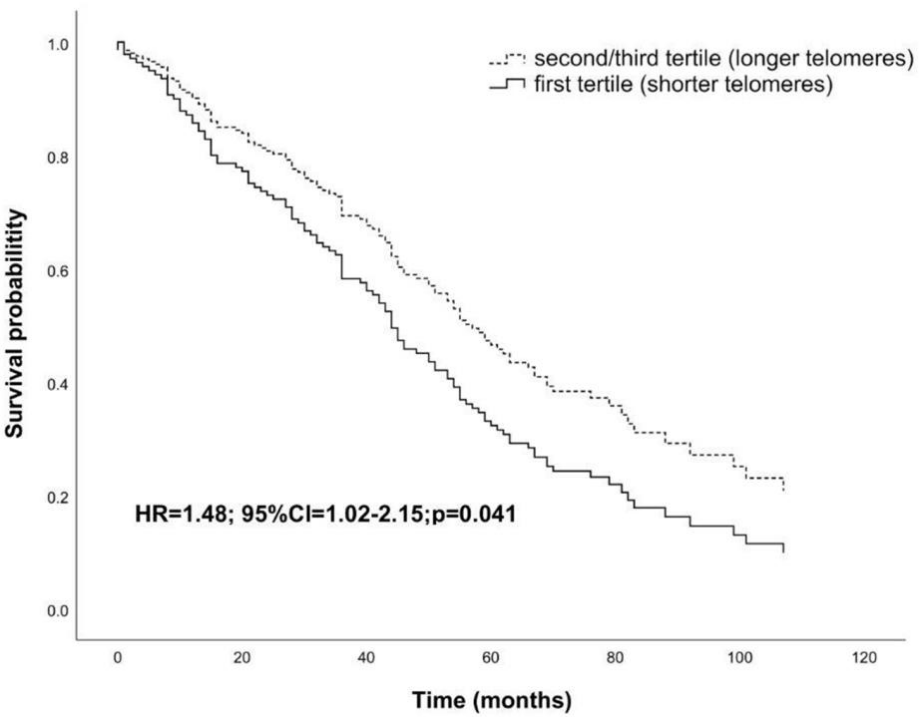
**Survival functions relative to carriers of shorter telomeres (first tertile) vs carriers of longer telomeres (second/third tertile) in the follow-up sample after seven years from the baseline visit.** The Cox regression was adjusted for age and sex. Time is expressed in months, where zero is considered the time of recruitment. HR value, confidence interval, and p-value from Cox regression analysis are reported inside the figure.

Interestingly, the LTL measured after the 7 years of follow-up was found to be inversely correlated to survival (HR= 1.48; 95% CI=1.02-2.14; p=0.041; Figure 4).

Moreover, we calculated the difference in LTL between the first and second sampling (delta-T/S) and then we divided the sample into subjects with higher attrition (first tertile) and subjects with minor attrition (second/third tertile). As it can be seen in Supplementary Figure 3, although the sample was quite small, was found a greater telomere attrition in subjects with longer telomeres at baseline [mean (SEM) = 1.37 (0.12)] as compared with those with shorter telomeres [mean (SEM) = 0.84 (0.61)] (p < 0.0001).

**Figure 5:**
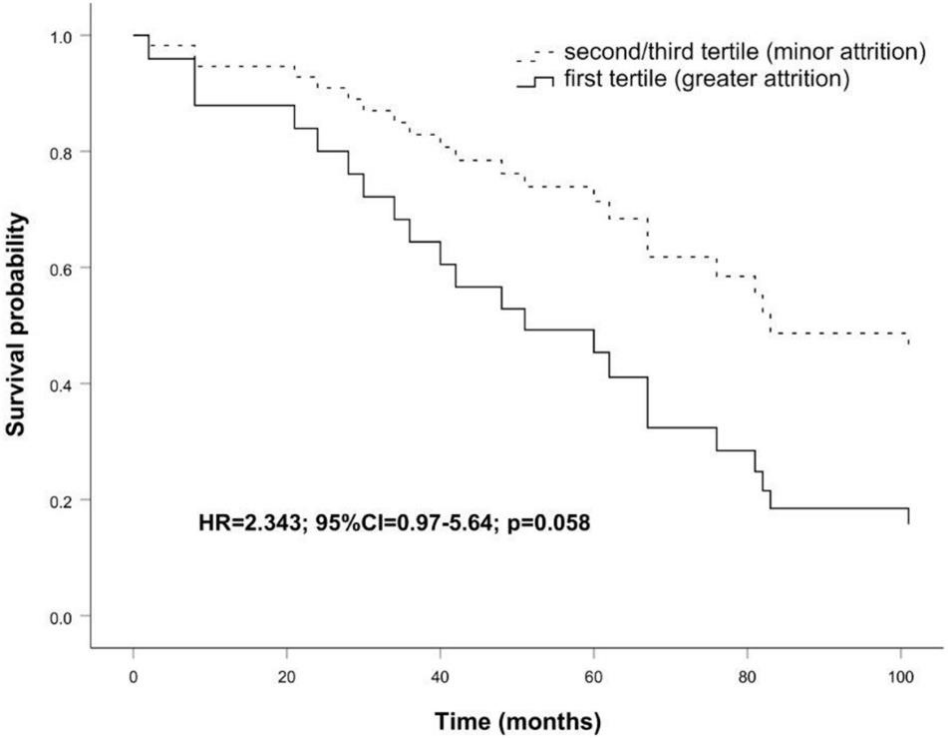
**Survival functions relative to carriers of greater attrition (first tertile) vs carriers of minor attrition (second/third tertile) conducted on the follow up sample.** The Cox regression was adjusted for age and sex. Time is expressed in months, where zero is considered the time of recruitment. HR value, confidence interval, and p-value from Cox regression analysis are reported inside the figure.

Also, survival analysis (Figure 5), obtained with sex and age as covariates, showed that subjects suffering during the follow-up a minor telomere attrition tend to live longer than those having higher telomere attrition in the same period (HR=2.343; 95% CI=0.97-5.64; p=0.058).

## DISCUSSION

One of the most debated questions in gerontological research is if telomere length can be considered a predictor for a long and healthy life. Problems in results’ interpretation for different studies come from the heterogeneity of factors affecting LTL (influenced by genetics, epigenetics, and environment), from the lack of proper controls and, last but not least, from the availability of follow-ups of both LTL and survival for recruited samples.

This study was carried out in Calabria, a region of Southern Italy. It should be noted that Calabrians, as reported elsewhere, represent a population quite different with respect to other populations studied so far (e.g., Danes or Caucasian-Americans) on this matter [8, 13, 14, 16]. In fact, Calabrians are a homogeneous population (very low levels of immigration have occurred in the last centuries), with a largely rural society where Mediterranean diet was largely predominant until a few decades ago, that is for most of the lifetime of the subjects who are now old adults. Indeed, previous studies comparing the aging population of Calabria to other Caucasian populations have noted specific features of this population [47–49].

The present data, observed in a sample of individuals aged 65-106 years followed for 10 years, provide some new clues on the complex relationship between LTL survival and lifespan.

Considering the whole sample, we did not find association of LTL with mortality. However, by analyzing LTL distribution as a function of age we found a curve which progressively decrements up to an inflection point around the age range 83 to 92 years, after which LTL increases slowly and levels off thereafter. This distribution turned out to be statistically highly significant (p<0.0001) and has many points in common with survival curves defining the Gompertz law, which describes the lifetime long pattern of acceleration and deceleration of mortality [50, 51]. The most recent analysis of Gompertz curves with data on modern populations have shown that survival curves of Western human populations show relatively little mortality during the first period of life, while the number of survivors declines approximately exponentially thereafter, usually after 70 years of age [52, 53]. Then, after a deep slope of the survival curve, corresponding to a dramatic increase of mortality chance, mortality attenuates and eventually levels off [52, 54, 55]. This trend of the survival curve has been mainly ascribed to the impact of selective survival in heterogeneous populations [55]. That is, most of the subjects within a population go through a biological decline which leads to death between 70 and 85/90 years of age. Those who are equipped to survive (due to genetic, biological or environmental factors) go through this age window and then after 90 years of age we have a selected population well equipped to live longer, and this explains mortality deceleration [54, 55]. The trend of LTL as a function of age in our sample population parallels the survival function reported for western societies and may have similar explanation. Indeed, we can hypothesize in subjects below 70 years of age a great heterogeneity of LTL due to genetic and lifestyle factors. As mentioned above, after this age an important increase of attrition has been noted [40]. This might be related to the general decline of the homeostasis which leads to the decline of most of the cellular and organismal functions predisposing to frailty and to the increase of oxidative stress and inflammation, which are among the main factors favoring telomere attrition. As reported above, we identified a temporal window, the interval of 83–92 years, in which subjects with shorter LTL had an increased mortality risk during follow-up than those with longer LTL. It is likely that within this age window we have subjects who have undergone significant telomere attrition and have then exposed the parts of the telomere predisposing to senescence [56]. On the other hand, within this age window we have also subjects who did not lose their homeostasis capacity and did not undergo such attrition. Thus, the inverse correlation between LTL and survival in this age window does reflect the heterogeneity due to subjects who underwent a significant attrition and those who did not yet. After this age window, we find mainly selected subjects and the older is the age the highest will be the selection and then the lowest the attrition undergone. We may summarize the whole process by stating that we see, at the population level, two phenomena going on after 70 years of age: accelerated frailty correlated with attrition and selection. In support of this hypothesis, we have the data from subjects observed after seven years from the first recruitment. We observe no correlation between LTL and mortality at the baseline, but an inverse correlation when we consider the observations taken after seven years of follow-up. This might be due to a significant attrition in some subjects, who eventually die soon after, while no attrition, or a limited attrition is experienced by the others, who survive longer. Consistently, we find a direct correlation between attrition occurred in the seven years between the first and the second observation and mortality. Also, it is very interesting that individuals with the longest telomeres at baseline experienced the greatest amount of shortening over time, a result which is in accordance with other studies [57, 58]. This telomere length-dependent attrition rate could reflect selection effects whereby cells with short telomeres and high attrition rate are less likely to survive than cells with longer telomeres. Similarly, it is possible that subjects with shorter telomeres experiencing stronger attrition have a higher mortality rate and then the phenomenon we observe may be due to such demographic selection. On the other hand, this phenomenon could reflect that telomerase, as reported in model organisms, seems to preferentially act, in stem cells, on the shortest telomeres [59, 60]. In this case, we can hypothesize the action of telomerase within the cells progenitors of white blood cells.

We are aware that our study suffers from some limitations mainly represented by the relatively small sample size, regarding the number of subjects both in the 83-92 age class and in the sub-sample of individuals with the second LTL measurement. On the other hand, it should be considered the peculiarities of the samples, collected within a homogenous population, and followed up for a long period. Thus, although additional studies in a larger sample will be needed to strengthen the findings of our study, we are confident that our observations, based on a sound sampling strategy, are revealing a phenomenon that will deserve to be deeply analyzed.

In conclusion, the present study, by analyzing the trend of LTL as a function of age in a population sample ranging between 65 and 106 years of age, suggests a parallel between demographic mortality curves and LTL, hypothesizing that a generalized LTL attrition after 70 years of age is due to the organismal decline affecting the majority of the population. Such generalized attrition may lead, on turn, to exacerbate senescence in these subjects and predisposing them to high mortality risk. On the other hand, the subjects with better physical conditions, experience a lower attrition (or postpone it) and a delayed senescence, contributing to the deceleration of mortality curve which has been observed in modern societies. Should this hypothesis be true, telomere length may be predictive at the population level for survival in heterogeneous populations, such as the population within 83 and 92 years of age, but not in either unselected population, such as the population under 70 years of age, or selected populations, such as the population over 92 years of age.

## MATERIALS AND METHODS

### Subjects

The sample analyzed included 516 unrelated subjects (240 men and 276 women, aged 65-106 years; median ages 80.54+10.87). All the subjects were born in Calabria (Southern Italy) and recruited in the entire region through several campaigns, as previously reported [47]. Vital status was traced after a mean follow-up time of approximately 10 years through the population registers of the municipalities where the respondents lived. A sub-sample of 36 subjects was re-recruited after 7 years and the vital status was verified after about 10 years from the last visit. At baseline, all subjects were free of the major age-related pathologies.

### Ethics statement

Investigation has been conducted in accordance with the ethical standards established in the Declaration of Helsinki and has been approved by the authors' institutional review board. Each subject, before the visit, signed an informed consent, for the permission to collect blood samples and usage of register-based information for research purposes.

### Leukocyte telomere length (LTL)

The average length of telomeres was measured by Real-Time PCR quantitative analysis (qPCR), by using the MiniOpticon Monitor Real Time PCR System (Bio-Rad, 48-wells format). This method allows to measure the number of copies of telomeric repeats (T) compared to a single copy gene (S), used as a quantitative control [61]. We used the modified protocol described by Testa and colleagues [62]. For the PCR reaction, 5 μl of DNA with a concentration of 3 ng/μl (15 ng in total) and 15μl of mix (containing the specific primers for telomeres (T) and control gene (S), the PCR reagents and the SYBR green dye for the detection of the fluorescence) was added in each well. Concentrations for telomere and 36B4 PCR primers sequences and the thermal cycling profile were as reported by Testa and coworkers [62]. In addition, two standard curves (one for 36B4 and one for telomere reactions) were prepared for each plate by using a reference DNA sample (Roche, Milano, Italy) diluted in series by 1.68-fold per dilution in order to produce 6 concentrations of DNA ranging from 30 to 2 ng in 5μl. The telomere and single-copy gene (36B4) were analyzed on the same plate to reduce inter-assay variability. R^2^ and amplification efficiencies varied between 0.982 and 0.998, and 95.2 to 101%, respectively. More than 20% of samples were blindly replicated on different plates to assess T/S measurement reproducibility. The inter assay coefficient of variation was <8%. A calibrator sample (Roche, Milano, Italy) (3ng/ul in 5 μl) was included in each plate. Measurements were performed in triplicate and reported as T⁄S ratio relative to the calibrator sample to allow comparison across runs.

### Statistical analyses

The regression line with a cubic slope was calculated using the least squares method and a constant. The associations between T/S ratio and survival were investigated using Cox regression analysis. T/S ratio was analyzed as a categorical variable. The categorical variable was derived as the tertiles of T/S ratio; the group with the shortest T/S was used as the reference category. To estimate the effect of the telomere length on survival, we evaluated survival after 10 years from the baseline visit. Subjects alive after the follow-up time were considered as censored, and this time was used as the censoring date in the survival analyses. Hazard ratios (HR) and 95% confidence intervals (95% CI) were estimated by using Cox proportional hazard models considering age and sex as confounders variables. For a sub-sample of 36 subjects, we calculated the telomere attrition by difference of T/S ratio between the first visit and the second, after about 7 years. Also, in this case the categorical variable was obtained as the tertiles of delta T/S; the group with the major attrition (first tertile) was used as the reference category. A P value < 0.05 was considered statistically significant. All statistical analyses have been performed using R system (version 3.6.2).

### Data sharing statement

The data that support the findings of this study are available from the corresponding author upon request.

## Supplementary Material

Supplementary Figures
