# Supplementary material for: Telomere length as a function of age at population level parallels human survival curves
Source: Aging (Albany NY). 2021 Jan 11;13(1):204–18. doi: 10.18632/aging.202498 (PMC7835060; doi:10.18632/aging.202498)
Supplement: Supplementary Figures [file aging-13-202498-s001.pdf]

SUPPLEMENTARY FIGURES

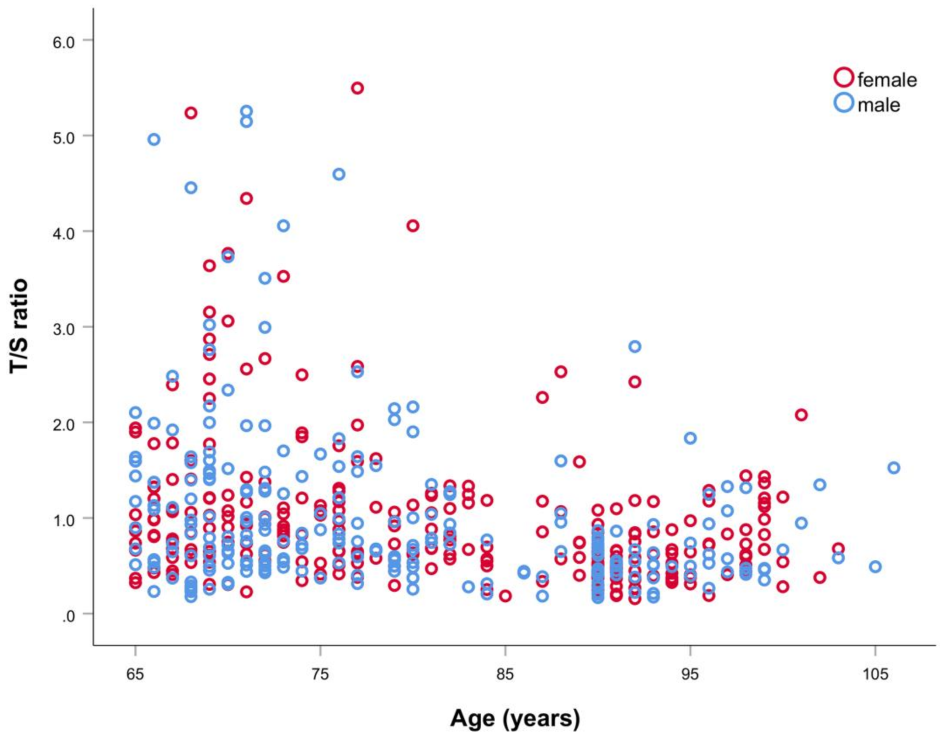

Supplementary Figure 1. Scatterplot showing the relative Leukocyte Telomere Length (LTL) expressed as T/S ratio in males and females.

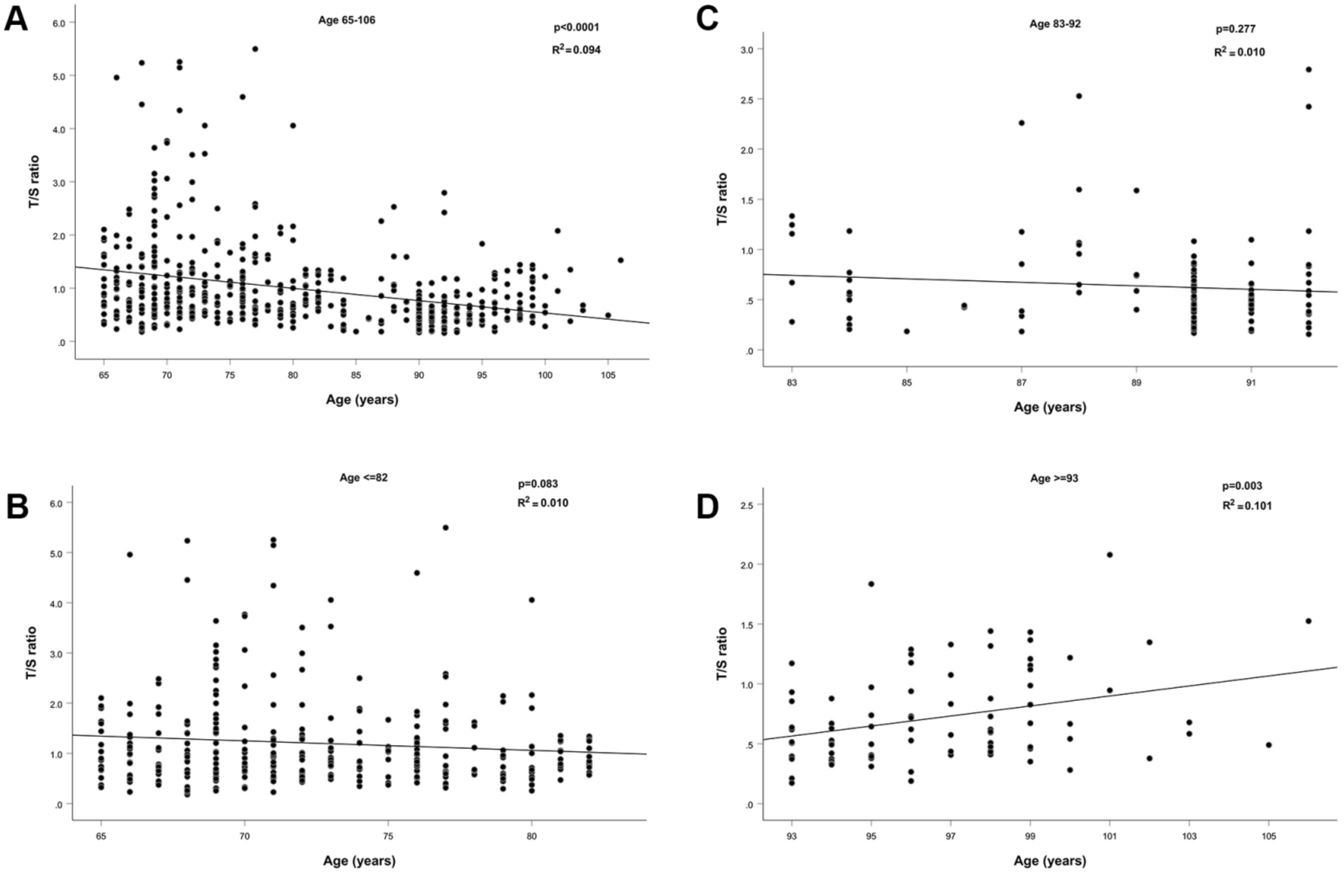

Supplementary Figure 2. Scatter plots showing the relative Leukocyte Telomere Length (LTL) expressed as T/S ratio in the whole sample (**A**) and in the sample divided in age ranges: from 65 to 82 years (**B**), from 83 to 92 years (**C**) and from 93 to 106 years (**D**). The linear regression line fitting the data, the R^2 and p-value are shown in each panel.

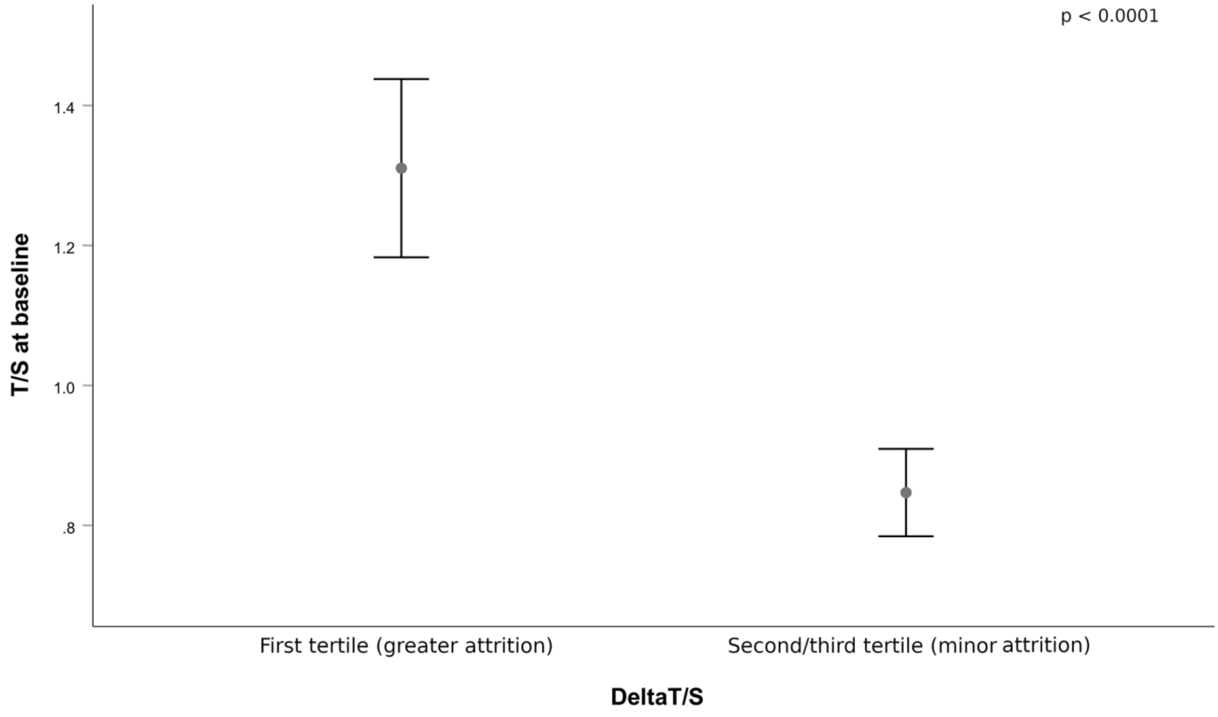

Supplementary Figure 3. T/S values at baseline of the subjects within the first tertile (greater telomere attrition) and second/third tertile (minor telomere attrition) of the delta T/S values. The data are reported as mean ± standard error of the mean (SEM) and p-value computed by t-test.
